# Disinfectants

**Published:** 1887-07-23

**Authors:** 


### Disinfectants.

Summer is the season for the free use of disinfectants. The rapidity of decomposition is generally directly proportionate to the temperature. Lately the heat has been excessive, and all kinds of wholesome and unwholesome smells have assailed the nostrils. Few people question the value of disinfectants—none who have had much experience of them. All kinds of pipes should now be well flushed, and frequently and freely disinfected. The best disinfectant is fresh air. That cannot always be forced into drains and soil-pipes in sufficient quantity to really disinfect. There are several manufactured articles now in use which can be conveniently substituted for fresh air where that is not really available, but in general windows and doors should be thrown wide open night and day, and manufactured disinfectants should have a complementary place. Among the most convenient, pleasant, and effective may be mentioned Sanitas, prepared by the Sanitas Company. Several samples have been sent to us, which we have tested in a variety of practical ways, and with great satisfaction. The liquid sanitas is suitable for use in flushing drains, and the sanitas soap is excellent for chronic eczemas, sweating and blistering feet, and other troubles incidental to the summer season.

